# Basic Concepts in the Taxonomy of Health-Related Behaviors, Habits and Lifestyle

**DOI:** 10.3390/ijerph10051963

**Published:** 2013-05-13

**Authors:** Luis Salvador-Carulla, Federico Alonso, Rafael Gomez, Carolyn O. Walsh, José Almenara, Mencía Ruiz, María José Abellán, eVITAL group

**Affiliations:** 1 Centre for Disability Research and Policy, Faculty of Health Sciences, University of Sydney, 75 East St. Lidcombe, NSW 2141, Australia; 2 Spanish Association for Research of Healthy Aging (Asociación Española para el Estudio Científico del Envejecimiento Saludable, AECES), Calle Infante Don Fernando 17, 29200 Antequera (Málaga), Spain; E-Mails: falonso@longevidad.org (F.A.); rgomez@longevidad.org (R.G.); 3 Boston Combined Residency Program in Pediatrics, Boston Children’s Hospital and Boston Medical Center, 300 Longwood Ave, Boston, MA 02115, USA; E-Mail: carolyn.o.walsh@gmail.com; 4 Faculty of Nursing and Physiotherapy, University of Cadiz, Av. Ana de Viya 52, 11009 Cádiz, Spain, E-Mails: jose.almenara@uca.es (J.A.); mariajose.hervas@uca.es (M.J.A.); 5 Scientific Association PSICOST, Plaza de San Marcos 6, 11403 Jerez de la Frontera (Cádiz), Spain; E-Mail: mencia.ruiz@gmail.com; 6 eVITAL group, Spanish Association for Research of Healthy Aging (Asociación Española para el Estudio Científico del Envejecimiento Saludable, AECES), Calle Infante Don Fernando 17, 29200 Antequera (Málaga), Spain

**Keywords:** health behavior, health habits, life style, longevity, classification, health terminology, taxonomy

## Abstract

*Background*: Health-related Habits (HrH) are a major priority in healthcare. However there is little agreement on whether exercise, diet, smoking or dental hygiene are better described as lifestyles, habits or behaviors, and on what is their hierarchical relationship. This research is aimed at representing the basic concepts which are assumed to constitute the conceptual framework enabling us to interpret and organize the field of HrH. *Methods*: A group of 29 experts with different backgrounds agreed on the definition and hierarchy of HrH following an iterative process which involved framing analysis and nominal group techniques. *Results*: Formal definitions of health-related behavior, habit, life-style and life-style profile were produced. In addition a series of basic descriptors were identified: health reserve, capital, risk and load. Six main categories of HrH were chosen based on relevance to longevity: diet/exercise, vitality/stress, sleep, cognition, substance use and other risk. Attributes of HrH are clinical meaningfulness, quantifiability, temporal stability, associated morbidity, and unitarity (non-redundancy). Two qualifiers (polarity and stages of change) have also been described. *Conclusions*: The concepts represented here lay the groundwork for the development of clinical and policy tools related to HrH and lifestyle. An adaptation of this system to define targets of health interventions and to develop the classification of person factors in ICF may be needed in the future.

## 1. Introduction

Although the first recognition of the importance of promoting healthy lifestyles could be traced back to Baron Horder’s speech in Leeds in 1937 [1], it was not until 1974 that the Lalonde report in Canada provided a specific policy agenda on the area of “lifestyle” [2]. This report was followed by increasing interest on the influence of lifestyles on outcomes and population mortality, and the incorporation of health-related habits (HrH) to a series of national periodical health surveys [3,4,5]. Countries have monitored temporal trends in HrH and related illnesses and health indicators, and have launched ambitious plans to improve the HrH of the population [6].

Despite these efforts, there doubtless exists a gap between the importance of lifestyle in the health of individuals and the capacity of physicians to evaluate and intervene in the lifestyle of an individual patient [7]. Physicians lack sufficient training in helping patients achieve behavior change, and the available tools are limited [8] or directed at specific diseases [9], as opposed to being holistic. Intervention programs on health behaviors and lifestyle [2,10,11] and the growth of online systems for the evaluation of HrH are important early steps in the integration of intervention strategies for HrH into primary care [12]. However, in order to increase the impact of these numerous efforts moving forward, it is necessary to reach a common understanding of the terminology of definitions used in the field and of how lifestyle, HrH, and behavior can be classified in a hierarchical way. The International Classification of Functioning, Disability and Health (ICF), developed by the World Health Organization (WHO) in 2001, mentions HrH and lifestyle as key components of the personal factors of functioning, but does not provide definitions or a classification of these factors. The lack of a preliminary taxonomy impedes both the incorporation of an adequate form of HrH into medical information systems [13,14], and the integration of intervention strategies into both clinical practice and into public health policy. A parallel example showing the importance of health classification systems is the recent development of a taxonomy of medical errors in primary care [15], which allows for the incorporation of related data into systems of health information [16].

The Spanish Association for the Scientific Study of Healthy Aging (*Asociación Española para el Estudio Científico del Envejecimiento Saludable*, *AECES*), has recently developed a preliminary taxonomy and a related toolkit for the evaluation of HrH applicable in primary care [17]. The objective of this paper is to represent the different aspects of the basic concepts (metacategories, categories and other relevant concepts) which are assumed to constitute the conceptual framework enabling us to interpret and organize the field of Health-related Habits for clinical practice, research and health policy.

## 2. Methods

Between 2004 and 2010, AECES conducted a framing analysis [18] of the field of health-related habits, created a taxonomy and an online toolkit (eVITAL) for evaluation of HrH, and analyzed the feasibility of eVITAL according to expert opinion and data from a demonstration study of 11 middle-age subjects, healthy by self-report, from whom written informed consent was obtained. A panel of experts in different health professions and with distinct areas of specialized knowledge, followed an iterative process to produce this system including five working sessions of a nominal group with six members and four focus groups consisting of 29 experts. The detailed protocol for this study is available elsewhere [19]. 

The current article focuses on the process of using the conceptual framework established by the expert group to produce formal and operational definitions of the basic concepts that include the following “metacategories” (categories that contain other categories, classified as indicated by the category name): Health-related Behavior, Health-related Habit, and Health-related Lifestyle. The result was incorporated into the assessment system of HrH that was then evaluated by the focus groups, computerized, and revised along with the taxonomical model and its hierarchical classification. 

### Taxonomical Model

The expert panel adapted the taxonomical model of the ICF [20] and a series of complementary WHO documents which provided definitions of concepts related to health promotion, healthy aging and health habits [21,22,23]. Also integrated into this model were concepts from the multilevel person-centered diagnosis model [24] and the transtheoretical model of stages of change and the related multibehavioral change model [25,26,27]. 

A construct is a complex, non-discrete entity or class. “Non-discrete” refers to the theoretical or intangible nature of constructs; by definition, unlike quantifiable discrete entities such as height, temperature, or disease states such as breast cancer and HIV infection, constructs are not directly measurable. The ICF distinguishes between constructs, domains and dimensions. These terms provide a preliminary understanding of the categories and metacategories described in the classification system and should be followed by the development of taxonomic hierarchy by formally defining the relationships (e.g., levels of superiority or subordination) of the different entities to compose successively larger groups into which the class is represented. It can be described and depicted using associative phrases such as <is a>, <is part of>, <is a type of> and <is composed of> [13].

Because of the complexity of constructs, it is helpful to divide them conceptually (<is composed of>) into hierarchical levels of subentities or children categories, each of which occupies a specific niche within the construct. In decreasing order of size and complexity, the subentities are domains, dimensions, subdimensions, and items. Within the ICF model, domains could be regarded as the subentities that make up (<are part of>) the construct, and are defined as a specified order of related ideas, materials, or knowledge. Each domain can itself be divided into several dimensions, or related second-order subclasses. For example, the construct “lifestyle” is a metacategory that contains several domains (work, health) whereas its domain “health*-*related lifestyle” can be described using several basic dimensions (physical, psychological, social). These subentities should be clearly defined, such as by presence or absence using a pre-set threshold. At the same time “health-related lifestyle” can be represented as the metacategory that contains all the health-related habits (domains). By creating operating definitions of constructs in terms of the hierarchical relationship of its well-defined domains and dimensions, the intangible construct becomes quantifiable. This approach has been previously used to describe other complex health constructs such as functional dependency [28].

## 3. Results

**Figure 1:**
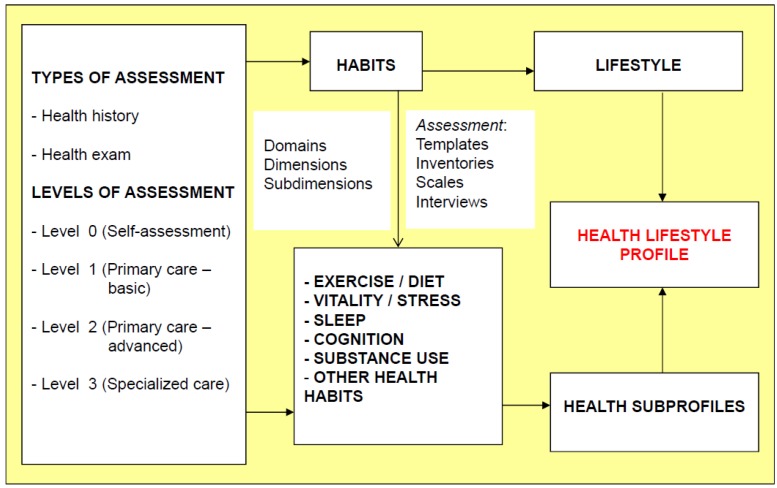
Conceptual framework of the assessment schema, health-related habits, and health lifestyle profiles of the eVITAL toolkit. (The full eVITAL taxonomy is available in Spanish at www.longevidad.org, Alonso *et al.* [19] and Salvador-Carulla L. *et al.* [17]).

The panel of experts adopted the tenets of longevity medicine, in which major endpoints include mortality, disability-adjusted life years, and years lived without disability within a life-span perspective. Using these criteria the experts included 6 HrH in the taxonomy and in the eVITAL clinical evaluation system: diet/exercise, vitality/stress, sleep, cognition, substance use, and other health habits (Figure 1). The granularity of this preliminary taxonomy includes three subdomains, 43 dimensions and 141 subdimensions [17]. An additional domain of “health descriptors” was included in the eVITAL due to the clinical significance of the social and demographic determinants in understanding HrH, even though they are not part of the hierarchy and the HrH taxonomy tree.

The group of experts took a holistic and integrative perspective to reach consensus on hierarchical definitions of health-related behaviors, habits, lifestyles and the related health lifestyle profiles. To define the concepts a bidirectional scale including both negative and positive aspects was created at three increasing levels of complexity—behavior, habits, and lifestyle—each of which contributes to an individual’s overarching “health lifestyle profile”. The concepts were arranged hierarchically using a tree structure using letters for the domains (health habits/subprofiles) and numbers for the dimensions and subdimensions. 

### 3.1. Basic Concepts

“Behavior” is an umbrella term that describes both the aggregate of responses to internal and external stimuli and the specific observable conduct of an individual. Here this term is used in the latter meaning. This taxonomic hierarchy describes the behaviors related to health as <part of> the health-related habits, which themselves are <part of> the lifestyle of an individual. Each habit is <related to> a particular health subprofile, as described below. Health-related behaviors, habits and lifestyle are part of the person context within the framework of the WHO model of human functioning and its classification (International Classification of Functioning—ICF). In the ICF/WHO model personal factors “are the particular background of an individual’s life and living” [20]. They comprise features of the individual that are not part of a health condition, such as social and demographic factors (gender, race, age, education, profession) and individual psychological characteristics, such as lifestyle, habits, upbringing, coping styles, overall behavior pattern and character style. Hence, individual functioning is influenced by personal contextual factors that are different from environmental factors. Within the person-centered model HrH are divided into those that contribute to positive health and those that contribute to ill health; they are included in the class “internal health determinants or contributing factors” which also includes characteristics, attitudes, abilities, motivation to change and other components of an individual’s mental state [24].

#### 3.1.1. Health-Related Behaviors (HrB)

HrB are specific observable conducts for which there exist clinical, epidemiological, or social data that indicate at least a possible relationship with the promotion, protection, or maintenance of health (positive HrB, or HrB+), or with a greater susceptibility for, or risk of, poor health or illness (negative HrB, or HrB−). These behaviors are not isolated, but instead are associated with each other, forming patterns of behavior; most are done on a routine basis, which facilitates their evaluation and monitoring (see below). Importantly, there are some behaviors that individuals perform with the intention of improving health but for which there is no evidence of clinical benefit, such as homeopathic treatments, and others that are clearly harmful, for example refusing a childhood MMR vaccine because of its hypothetical relationship with autism. According to the expert panel these “health oriented behaviors” should not be considered HrB+. 

#### 3.1.2. Health-Related Habits (HrH)

HrH are a type of health determinants or contributors to health, characterized by acquired patterns of complex behaviors, related to basic instincts and motivations relevant for a health target (longevity in this case), that have been internalized to the point where they are carried out automatically. Habits are observable and they can be evaluated by a series of main descriptors or attributes (Table 1). Although HrH are typically stable, they may vary somewhat in different social contexts. The expert group provided two additional qualifiers of HrH based on the polarity and the stages of change model.

**Table 1:** Main Criteria (Attributes) for typifying Health-related Habits in the assessment of the Health Lifestyle Profile (*).

Criteria	Specifications
**1. Clinical meaningfulness (Relevance to health)**	The habit is meaningful from a clinical and a public health perspective. There is published evidence in the scientific literature of the relationship of the habit with a priority target in health (e.g., longevity, disability, health promotion), which could be evaluated using standard indicators like mortality, disability-adjusted life years, and years lived without disability).
**2. Quantifiability (measurabity)**	Habits or behavior patterns linked with basic health functions can be broken down into a series of specific observable behaviors and/or behavioral patterns and are associated with health outcomes. By measuring specific behaviors and their combined pattern, one can determine an identifiable and objective pattern (e.g., watching more than three hours of television each day is a relevant indicator of sedentariness). It is possible to identify and describe the main components of each habit using specific standardized measurements.
**3** **. Temporal stability**	Habits or behavior patterns linked with basic health functions are stable over time and are performed routinely, as opposed to occasionally. The habit, its behavior pattern or a key observed behavior persists for more than 12 months.
**4. Associated morbidity**	The excess or the lack of use of the habit is associated to specific health conditions coded in the standard international health classification systems.
**5. Unitarity (non-redundancy) **	A habit is not redundant with other habits and cannot be better considered a dimension within another domain (*i.e.*, It fits as a category within the hierarchical classification)

* The habits identified by these criteria allow for the development of a profile of health-lifestyle that includes health-related behaviors and habits of each individual, which can be used to guide intervention strategies and prescriptions related to health habits.

**Figure 2:**
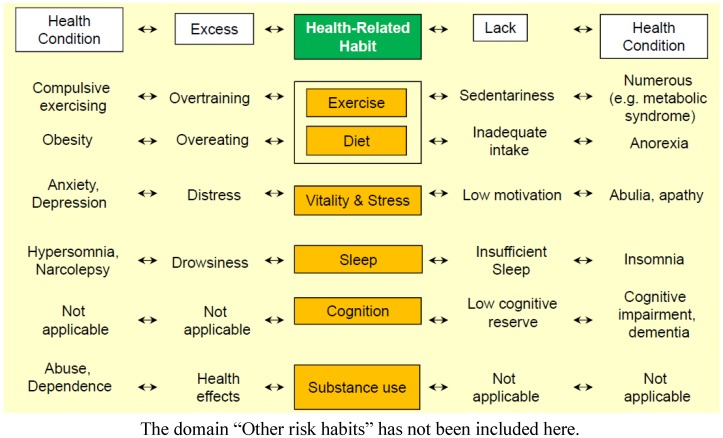
A directional model of Health-related Habits.

▪ Polarity: Habits can be classified into “positive health-related habits” (HrH+), complex behavioral patterns whose result is in increase in lifespan and in years lived without disability, and “negative health-related habits” (HrH−) associated with poor health and illness. In addition, two different patterns of polarity could be identified, when habits are represented in a continuum, unidimensional scale. From a clinical perspective some habits may be represented as bipolar/bidirectional within a continuum where proactive positive health (or enkrasia) [29] can be placed in the middle, and specific disorders associated either to excess or to lack of use are at the extremes (Figure 2). Two other HrH are better described as unipolar, ranging from optimal health to ill health related either to excess (substance use) or to lack of use (cognition).
▪ Stages of change: Of the six basic HrH, three are directly related to motivation to change (diet/exercise, substance use, and other risk habits), and three are less directly linked (cognition, vitality/stress, and sleep). The stages of change model developed by Prochaska [25] includes the stages precontemplation, in which an individual has no intention of changing within 6 months, *contemplation*, in which an individual intends to change within 6 months, preparation, in which small steps are taken with the goal of changing behavior within 1 month, action, in which specific changes have occurred within the past 6 months, maintenance, the stage of variable duration in which relapse is actively avoided, and *termination*, in which the individual is no longer tempted to return to the behavior [26], with *relapse* being a time in which an individual moves backwards from maintenance or termination to other stages. An assessment of an individual’s stage of change regarding a particular behavior raises the clinical utility of an evaluation of HrH by guiding clinicians toward interventions appropriate for a given stage and by predicting likelihood of behavior change [27,28,29,30,31].

Additionally, the consensus group established temporal limits for the evaluation of the stages “maintenance” (6 to 12 months) and “termination” (+12 months). The basic conceptual model of eVITAL appears in Figure 1; and the bidirectionality of the HrH and their relationships to specific health conditions are shown in Figure 2. 

The basic HrH are affected both by internal health determinants, like the trait of hostility for vitality/stress, and external ones, as when life events influence substance use and sleep. They can also be affected by specific health conditions, such as when cognition is adversely affected by the presence of depression. 

#### 3.1.3. Health-Related Lifestyle (HrL)

A lifestyle consists of the set of stable and quantifiable HrH, or patterns of behavior, of an individual. The lifestyle of each individual will combine HrH+ and HrH−; one example would be a person with high vitality/low stress who does a lot of intellectual activity, but who is sedentary. For this reason, unlike individual behaviors and habits, which can be considered either positive or negative for health, the bipolar differentiation of positive and negative HrL has limited utility in clinical practice. 

#### 3.1.4. Health Lifestyle Profile

**Figure 3:**
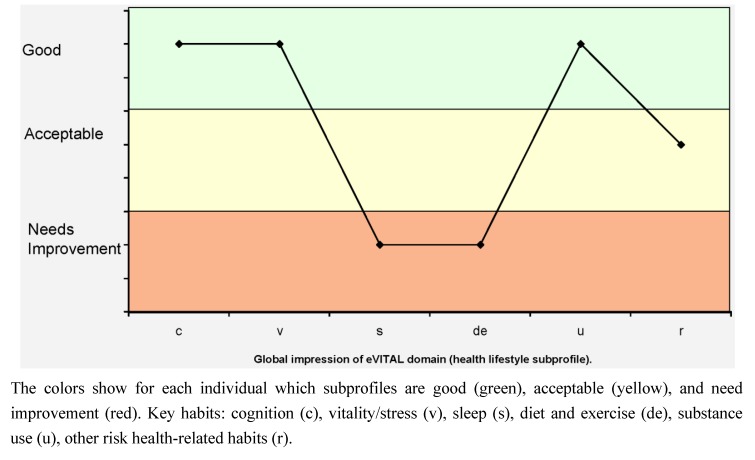
The “Health Lifestyle Profile” graphically represents the global impression of each eVITAL domain (health subprofile).

A health lifestyle profile is a graphical representation of the data describing an individual’s habits and lifestyle, obtained from a variety of questionnaires and clinical tests (see Figure 3). The expert panel decided to use a global impression of every HrH for routine practice and to increase its clinical utility by integrating information about other health determinants and pathology associated with each habit. Because of this, once the six basic health habits were identified, the panel described six “health subprofiles” related to these habits. In addition to behavioral patterns, these include internal and external health determinants and specific health conditions directly related with each habit. The six main subprofiles of the classification scheme correspond with the HrH: cognition (c), vitality/stress (v), sleep (s), diet and exercise (de), substance use (u), and other health-related (risk) habits (r). 

### 3.2. Other Relevant Concepts

#### 3.2.1. Health Reserve and Capital

Health “reserve” or capital refers to the series of physical/biological, functional, cognitive, psychological, and social resources to which a person can turn when additional demands are placed on the body by illness, accidents and injuries, and other stressors, or when the body’s normal abilities are diminished by age and the passage of time. 

The classification scheme employs the concept of reserve to define specific subcategories in the domains “cognition” and “vitality/stress” to describe the positive poles of these subprofiles both conceptually and in graphical representation. “Cognitive reserve” refers to the part of our cognitive resources which only come into play when the brain receives excessive demands or when age or pathology reduces our cognitive capacity. This reserve can compensate for organic injuries except those from major lesions, such as brain atrophy and very large or critically placed cerebral infarcts, and even in these cases can play a fundamental role in an individual’s adaptation and rehabilitation. 

In the subprofile of vitality/stress, “psychosomatic reserve” consists of a series of characteristics that are associated with a lower risk of medical problems in general and on specific area (e.g., cardiovascular disease): the tendency toward good-naturedness *versus* aggressiveness and restraint *versus* impulsivity, and absence of the Type A personality. Reserve or “social capital” refers to trust between people, mutual interactions and obligations and engagement in civic activities through social networks and volunteer associations. Measurements of social reserve consider these trust- and obligation-building social resources and economic and cultural groups at the level of the family, neighborhood, and community. The social capital cultivated through these relationships, expectations and obligations helps individuals to deal with challenges more effectively. 

Health reserve is subject to both gains and losses. A loss is a decrease in or failure to acquire abilities and reserve, due to lack of use, accidents, illness, or progressive decline. Gains in health reserve over time can be subclassified as net gains and compensatory gains, with net gains referring to abilities or cognitive skills that continue to develop and increase throughout life, and compensatory gains fully or partially counteracting the effect of loss or decline in other functions. 

#### 3.2.2. Health Risk and Load

The term “health risk” describes a series of physical/biological, functional, cognitive, psychological, and social characteristics that predispose an individual to ill health or that increase the vulnerability of an individual in the face of internal or external stressors. This term has been used in eVITAL in opposition to “reserve” to define the negative pole of the health subprofiles “cognition” and “vitality/stress” both conceptually and in graphical representation. 

“Health load” defines the decline of the physical/biological, functional, cognitive, psychological, and social resources of an individual over time and with exposure to internal and external stressors. The concept of “basic allostatic load” has been incorporated within the subprofile “vitality/stress” to describe a set of physiologic indicators of stress [32].

## 4. Discussion

The high terminological variability in prior work related to HrH hinders the synthesis of the existing evidence, the development of the HrH knowledge-base, and the design of comparable clinical and public health intervention strategies. For example, multimodal risk interventions for multibehavioral change [33] may be improved by the use of standard taxonomy of HrH. The standard taxonomy may facilitate the incorporation of HrH and related concepts into computerized health systems, which is a priority for health promotion moving forward [34].

Although definitions of HrH such as “sedentarism” have been recently developed [35], together with classification of both specific health-related behaviors [36] and the behavioral change intervention techniques [37], to the best of our knowledge this is the first formal definition of the basic concepts to be incorporated to a hierarchical taxonomy of HrH and lifestyle primarily intended for use in clinical practice and public policy. 

The resulting taxonomical system differs in several important aspects from prior models. For example, in the past “diet and exercise” have been typically categorized separately in spite of their early combined categorization at the Lalonde report in 1974 [2]. The importance of considering both categories in a common domain to design “multiple health behavior interventions” has been recently outlined both for public policy and clinical practice [38]. Furthermore “multiple health behavior” patterns are better understood when defined as HrH. On the other hand other habits such as sleep, cognition or non-substance use risk habits are seldom consider when listing habits and life-style. Sex-related behaviors and habits have not been listed as main domains at eVITAL, as experts considered that these behaviors did not have a first order effect on longevity and are mediated by other domains as other health risk behaviors and vitality/stress where they have been listed in the eVITAL preliminary taxonomy [19]. Other contributions of the eVITAL typology are: (i) the distinction between different levels of complexity in the proposed hierarchy (individual health-related behaviors, habits, lifestyle and lifestyle profile), (ii) the use of a series of related concepts to refine the lower levels of the taxonomy (health reserve, capital, risk and load), and the identification of specific attributes and qualifiers to operationally describe the main habits. 

Our classification of the six domains of HrH was guided by the transtheoretical model of motivation to change [25,26]. This model and the related multibehavioral change approach [27] has been applied to a series of HrH such as smoking, alcohol abuse, physical inactivity, poor diet, stress or sun exposure, but not to cognition, vitality, or sleep, following an integrative approach.

## 5. Conclusions

The consensus on the health terminology and on the hierarchy of HrH is necessary to develop a practical taxonomy. Health-related Lifestyle is here represented as the parent category of Health-related Habits and Health-related Behaviors are their children categories. Our work used longevity as an endpoint and had primary care as its main target. Although it took a holistic and integrative focus regarding HrH, and it coincides with the current perspective of WHO on aging [39], it is important to note that the resulting taxonomy may not be applicable to other endpoints such as disease (as in ICD) [40], functioning and disabilities (as in ICF) or public health promotion. On the other hand the categories defined here and the tree taxonomy described at eVITAL do not represent a formal ontology system. 

To be fully generalizable this taxonomy tree needs further revision, testing and analysis using other end-points apart from longevity (e.g., specific health conditions or disease groupings). From the public health perspective the domain “Other” should be re-named and expanded to incorporate more health behaviors (e.g., behaviors related to personal care) using the directional approach already developed in other domains. In any case and due to the scarcity of previous qualitative research in this area, eVITAL concepts may contribute to a better and clinically meaningful categorization of HrH at the collaborative strategy developed by the International Health Terminology Standards Development Organisation (IHTSDO^®^) and the WHO Family of Classifications (WHO-FIC) [39]. It may also complement other initiatives addressed to develop a typology person factors within the WHO International Classification of Functioning (ICF) model. Geyh and colleagues have suggested specifications of psychological personal factors assessed in the literature and in the Spinal Cord Injury international database. They identified 8 cross cutting areas including “Patterns of experience and behavior”. Within this area they include the following “Lifestyle and habits”: physical activity, alcohol, tobacco, drug use, and health practices [41]. The granularity of this specification can be improved using the descriptions provided by eVITAL and other typologies of HrH. It may also contribute to develop the typology of behavioral targets in the future International Classification of Health Interventions (ICHI) [42] and in other taxonomies of behavior interventions [37].
